# You Fill My Heart: Looking at One’s Partner Increases Interoceptive Accuracy

**DOI:** 10.1037/cns0000110

**Published:** 2017-06

**Authors:** Lara Maister, Lilla Hodossy, Manos Tsakiris

**Affiliations:** 1 Department of Psychology, Royal Holloway, University of London

**Keywords:** interoception, heartbeat perception, self-awareness, emotion regulation, romantic partner

## Abstract

The integration of external and internal bodily signals provides a coherent, multisensory experience of one’s own body. The ability to accurately detect internal bodily sensations is referred to as interoceptive accuracy (IAcc). Previous studies found that IAcc can be increased when people with low IAcc engage in self-processing such as when looking in the mirror or at a photograph of one’s own face. However, the way the self is represented changes depending on the context. Specifically, in social situations, the self is experienced in relation to significant others and not as an isolated individual. Intriguingly, in a relational context romantic partners can be used as social mirrors for one’s self. We here investigated whether directing one’s attention to romantic partners would enhance one’s IAcc, similar to the effect of self-face observation when the self is processed in isolation. During a heartbeat counting task, both concurrent self-face and partner-face observation improved accuracy in those with initially low IAcc; however, this improvement was significantly greater for the partner’s face. These results suggest that significant others may play an important role in determining the quality of one’s self-awareness. Given that high interoceptive awareness is linked to better emotion regulation, increased IAcc during partner observation is likely to have an adaptive role in maintaining stable and secure romantic relationships through greater emotion regulation.

The integration of external and internal bodily signals provides a coherent, multisensory experience of one’s own body, which fundamentally contributes to self-awareness (Ainley, Tajadura-Jiménez, Fotopoulou, & Tsakiris, 2012; Tsakiris, Tajadura-Jimenez, & Costantini, 2011). One channel of self-awareness is provided by “interoception,” which allows one to detect internal bodily signals. The ability to accurately detect these internal sensations is referred to as interoceptive accuracy (IAcc). One common method of measuring IAcc is to ask individuals to count their heartbeats, without taking their pulse (Schandry, 1981). Individuals show clear individual differences in their ability to perform this task, and these differences predict a wide range of other psychological processes. For example, individuals with high IAcc were found to experience emotions more intensely compared to people with low IAcc (Wiens, Mezzacappa, & Katkin, 2000). These differences in the ability to access emotions and bodily signals have distinct clinical implications. Researchers have reported links between high IAcc and panic disorder (Domschke, Stevens, Pfleiderer, & Gerlach, 2010) and between low IAcc and moderate depression (Dunn, Dalgleish, Ogilvie & Lawrence, 2007; Paulus & Stein, 2010), alexithymia (Herbert, Herbert, & Pollatos, 2011), psychosomatic disorders (Mussgay, Klinkenberg, & Rüddel, 1999), and anorexia nervosa (Pollatos et al., 2008).

Although these findings imply that IAcc can be considered as a trait-like characteristic with high test–retest reliability (e.g., Werner, Kerschreiter, Kindermann, & Duschek, 2013), recent studies have shown temporary state-like changes in IAcc as a function of different manipulations. For example, IAcc can be increased by viewing (a) one’s reflection in the mirror (Ainley et al., 2012; Weisz, Balazs, & Adam, 1988), (b) one’s own photograph (Maister & Tsakiris, 2014), or (c) words that are relevant to the self (Ainley, Maister, Brokfeld, Farmer, & Tsakiris, 2013). In all of these experiments, IAcc was enhanced when one’s attention was directed to self-related information, regardless of whether that information took the form of facial information or of verbal semantic information. Notably, across these studies, the changes in IAcc seem to appear only in individuals with low baseline IAcc (as measured when participants passively view a blank screen), which suggests a ceiling effect at the group with high IAcc and probably a less malleable self-representation in response to exteroceptive signals among participants high in IAcc (Tajadura-Jiménez & Tsakiris, 2014; Tsakiris et al., 2011). However, it is important to note that the distinction between state and trait components of IAcc have not yet been fully elucidated.

While these previous studies highlight that IAcc can be increased with self-focus, they only contrasted self-focus with attention directed to unfamiliar others. An important point, not considered by previous studies, is that self-representation can change depending on the social context (Markus & Wurf, 1987; Turner, Oakes, Haslam, & McGarty, 1994). In particular, when we are in the presence of significant others, such as romantic partners or close friends, a distinct self-experience, often referred to as the “relational self,” can be evoked (e.g., Andersen & Chen, 2002). This relational self-experience is an internal, first-person awareness, which differs from an objectified, external self-representation, which may be elicited in certain other social contexts, such as when being observed (Durlik, Cardini, & Tsakiris, 2014). Relational selves can be automatically activated not only by the presence of the significant other, but also merely by their imagined or symbolic presence, and can differ quite strikingly from the way the self is represented in isolated, more individualistic contexts (see Chen, Boucher, & Tapias, 2006, for review). Thus, if being in the context of a romantic partner activates a *relational* self-experience which is distinct from a nonrelational self-experience activated in other contexts, we may expect that focusing on the partner, rather than a representation of the self, may engender an enhancement of interoception in this case.

Importantly, the relational self not only contains conceptual information regarding “who we are” in the context of a significant other, but is also associated with a host of affective, behavioral and self-regulatory responses that are activated by the presence of a significant other. The self-regulatory aspects of the relational self are particularly relevant here. Researchers have found that individuals who exhibit good emotion regulation are able to establish and maintain closer and more stable romantic relationships by reappraising emotion-inducing events, rather than merely attempting to suppress their emotional reactions (Gross & John, 2003). Successful emotion regulation requires the awareness of one’s current emotional state, which in turn is closely linked to interoceptive awareness (Füstös, Gramann, Herbert, & Pollatos, 2013). For example, researchers have linked higher IAcc to more intense emotional experiences with respect to a given level of physiological arousal, accompanied by higher activation of underlying brain structures associated with emotional processing (Dunn et al., 2010; Pollatos, Traut-Mattausch, Schroeder, & Schandry, 2007; Wiens, 2005). Furthermore, individuals with high IAcc display more effective downregulation of affect (Füstös et al., 2013). Thus, given that self-regulation of arousal and emotional responses is so crucial in relational contexts, and a well-established link exists between emotion regulation and interoceptive awareness, one might expect an enhancement of interoceptive processing specifically in the presence of the partner, as a means of facilitating self-regulation.

In the current study, we aimed to answer two key questions: first, whether focusing on the partner may enhance interoceptive awareness in a way similar to focusing on the self, and second, whether previous findings regarding self-focus still hold when the self appears in the context of significant, close relationships. Because the experienced self at any one time is highly dependent on the social context, we predicted that the effects of self-focus on interoceptive awareness would also be context dependent. Specifically, given that a distinct, relational self is evoked when a romantic partner is imagined, symbolized or otherwise salient, we expected that focusing on an external representation of the partner (e.g., viewing the partner’s face) in this relational state would increase interoceptive awareness, in a way similar to focusing on an external self-representation (e.g., viewing one’s own face, or self-relevant words: Ainley et al., 2012, 2013; Maister & Tsakiris, 2014). This similarity in response may have an adaptive role, as high interoceptive awareness is linked to better emotion regulation and, in turn, more stable and secure romantic relationships.

Neuroscientific evidence lends indirect support to this prediction. Studies investigating the neural correlates of interoceptive processing have reliably identified the insula as having a key role in the awareness of internal sensations (Pollatos, Schandry, Auer, & Kaufmann, 2007). Intriguingly, the insula is not only activated when viewing one’s own face, but it is also activated when viewing a romantic partner’s face (Bartels & Zeki, 2000; Fisher, Aron, & Brown, 2005; Ortigue, Bianchi-Demicheli, Hamilton, & Grafton, 2007). Furthermore, the insula has also been implicated in emotion regulation (Giuliani, Drabant, Bhatnagar, & Gross, 2011). Accordingly, it may be the case that focusing on one’s romantic partner, as well as oneself, may enhance both interoceptive awareness and emotional regulation.

To test this prediction, we directly investigated how the interoceptive awareness of individuals in long-term romantic relationships changed when they viewed their partner or themselves, when they were placed in a relational context. We used a well-established heartbeat counting task to measure changes in IAcc compared with baseline during concurrent self-face and partner-face observation. The symbolic presence of the partner in the task was expected to elicit a context in which the partner was salient throughout the testing session, thus activating a relational self. As some findings suggest that adult attachment style affects both emotion regulation abilities (Diamond, Hicks, & Otter-Henderson, 2008; Feeney & Kirkpatrick, 1996) and the way the self is represented in relational contexts (Aron & Nardone, 2011), we also included a measure of attachment as a potential moderating variable.

## Method

### Participants

We recruited 32 undergraduate students from a U.K. university (23 female, *M*_age_ = 21.70, *SD*_age_ = 2.88) who reported that they had been in a romantic relationship for a mean duration of 31.56 months (*SD* = 27.97). According to a power analysis, our sample size for this study exceeded that needed to replicate the effect size reported in the most similar previous study (Maister & Tsakiris, 2014). We recruited participants on a volunteer basis. They received an information sheet explaining that the study aimed to measure heartbeat counting ability when looking at familiar or unfamiliar faces, and that two of the faces used would be their own and the face of their romantic partner. Both members of the couple were tested in case of seven couples (*n* = 14), whereas for 18 participants only one member of the couple was tested due to participant availability restrictions (our analysis controls for the potential effects of this difference between participants). All participants had a body mass index (BMI) within the normal range (*M*_BMI_ = 21.99, *SD*_BMI_ = 2.63). Our study has complied with American Psychological Association ethical standards in the treatment of the sample and was approved by the Department of Psychology Ethics Committee, Royal Holloway, University of London.

### Measures

Heart rate was monitored and recorded by a piezo-electric pulse transducer, attached to the participant’s nondominant index finger and measured their peripheral pulse (PowerLab 26T, AD instruments, Oxford, U.K.). IAcc was measured with the mental tracking method by Schandry (1981). Participants were given the following instructions before the task:

Please relax and concentrate on your body. Try to hear your heartbeat. At the “go” cue, start to silently count your heartbeats. You are not allowed to take your pulse while you do this. After the “stop” signal, you will be asked to report the number of heartbeats you have counted.

Participants were asked to attend to an image displayed on the computer monitor while they were counting their heartbeats.

To assess adult attachment quality, the 36-item Experience in Close Relationships–Revised Questionnaire (Fraley, Waller, & Brennan, 2000) was presented on the screen at the end of the testing session. This is a widely used measure of adult attachment, and possesses high test–retest reliability as well as high convergent and discriminant validity (Sibley, Fischer, & Liu, 2005). The questionnaire assesses two aspects of attachment: avoidance and anxiety. Participants are instructed to indicate how much they agree (on a Likert scale from 1 to 7) with an avoidance-related statement (e.g., “I prefer not to show a partner how I feel deep down”) or with an anxiety-related statement (e.g., “I often worry that my partner will not want to stay with me”). The order of items was randomized for each participant. The final scores of avoidance and anxiety were calculated by averaging scores on the related 18 items of each scale, taking into account reverse-scored items.

### Procedure

At the beginning of testing a photograph was taken of the participant’s face with a neutral expression using a Canon Legria HFR18 digital camera (Canon Ltd., Reigate, U.K.). In cases in which both members of the couple participated in the experiment, the self-photo was also used as the partner photo for their partner’s session. If a participant’s partner did not participate in the experiment then participants were asked to provide a passport-style photo of their partner showing their face and shoulders with a neutral facial expression. The use of pictures, instead of live and mirror observation, prevented individuals from picking up on subtle online cues of heartbeat, such as visually seeing their pulse in their neck in the mirror, which would facilitate performance in the heartbeat detection task.

After the photos were obtained, participants completed the heartbeat detection task, which was presented in the context of a within-subjects design. The task began with a 15-s training trial, which all participants completed successfully. The purpose of the training trial was to familiarize the participants with the task, and no feedback was given. After participants received written and verbal instructions, they completed the main heartbeat task. During each trial, an image was displayed on the computer screen. The image appeared immediately following the audiovisual start cue and remained on the screen until the stop cue appeared. This image was either the photograph of the participant’s own face (the self-face condition, mirror-reversed to match the participants’ most familiar view of themselves), the photograph of the partner (partner-face condition), or a black screen with a small fixation cross (baseline condition). The baseline condition was chosen to match the method used by previous studies to measure “trait” IAcc, which have shown good test–retest reliability (e.g., Werner et al., 2013). However, it is important to note that the distinction between state and trait components of IAcc has not yet been elucidated. Participants were reminded throughout the experiment to focus on the image shown for the entirety of each trial. Participants completed nine trials in total, three trials for each condition. Trials were presented in a random order for each participant and each trial was between 20 to 55 s in length. The total sum of duration for each of the three trials in each condition equaled 105 s. Participants were asked to type in the number of heartbeats they counted at the end of each interval. No feedback on their performance was given. During the heartbeat detection task, the experimenter was seated facing away from the participant, monitoring the physiological signal that was being recorded. Thus, we ensured that the participant was unlikely to pick up any nonverbal cues from the experimenter during the task, or to feel observed in any way. After finishing the heartbeat detection task, they completed the attachment questionnaire before being paid and debriefed.[Table-anchor tbl1]

## Results

Heartbeat traces were analyzed using LabChart6, which counted the number of R-wave-induced peaks in the peripheral pulse trace and calculated the average heart rate for each trial. Every heart trace record was visually examined for artifacts and the numbers of R-wave-induced beats were recounted manually if necessary. IAcc score was calculated for each trial with the following formula, where scores closer to 1 represent better IAcc:

$$1-\frac{\left|\mathrm{recorded beats}-\mathrm{counted beats}\right|}{\mathrm{recorded beats}}$$

IAcc scores for each trial were then averaged across each condition, to give a distinct IAcc score for each of the partner, self, and baseline conditions. IAcc scores did not significantly deviate from normality, Kolmogorov–Smirnov *D*(32) < 0.13, *p* > .200. Please see Table 1 for descriptive data.

For ease of interpretation, we calculated the *changes* in IAcc (IAcc_change_) by subtracting baseline IAcc scores from the self and partner conditions. These scores indicate the improvement or decline of IAcc during the observation of the self-face or partner-face. However, it is important to note that the same results were obtained when the initial scores, rather than change scores, were analyzed. Fitting a repeated-measures general linear model to IAcc_change_, with the within-subject factor of condition (SELF vs. PARTNER) and baseline IAcc inserted as a continuous between-subjects independent variable revealed the significant main effect of condition, *F*(1, 30) = 10.879, *p* = .003, η^2^ = .227, and a significant interaction between the condition and baseline IAcc, *F*(1, 30) = 7.172, *p* = .012, η^2^ = .151. This interaction indicated that the way in which our different conditions affected IAcc_change_ depended on individual participants’ baseline IAcc abilities.

**Figure 1 fig1:**
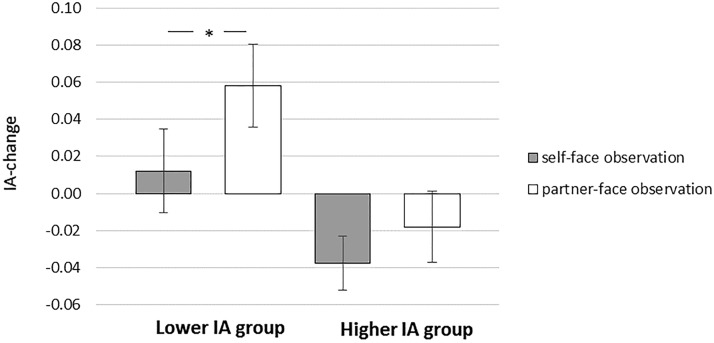
Graph showing the effects of self-face and partner-face observation on interoceptive accuracy (IAcc), for higher and lower IAcc groups. The dependent variable is the difference in IAcc from baseline. Positive values indicate an increase, and the negative values indicate a decrease in awareness. * *p* < .05, two-tailed. Error bars reflect standard error of the mean.

To investigate this interaction and to aid visualization of the results, we then performed a median split of baseline IAcc scores (following Ainley et al., 2012), whereby participants were assigned to either a lower or higher IAcc group depending on whether their baseline IAcc score was below or above the median (0.601). Paired-sample *t* tests on IAcc_change_ scores were then conducted between each condition in the lower and higher IAcc groups. A significant difference between IAcc_change_ in the self condition (*M*_self_ = .012, *SD* = .09) and the partner condition (*M*_partner_ = .058, *SD* = .10) was found in the lower IAcc group, *t*(15) = 2.265, *p* = .039, *d* = 0.478, but not in the higher IAcc-group, *t*(15) = 1.072, *p* = .301, *d* = .285 (see Figure 1). In the low IAcc group, two one-sample *t* tests demonstrated that IAcc_change_ in the partner condition significantly differed from zero, *t*(15) = 2.276, *p* = .038, *d* = .569, while IAcc_change_ in the self condition did not, *t*(15) = .537, *p* = .599, *d* = .134.[Fig-anchor fig1]

To examine whether arousal differed among conditions, a repeated-measures analysis of variance was carried out on average heart rate with condition (baseline, self-face, partner-face) as the within-subjects factor, but this did not reveal any significant differences between conditions, *F*(2, 62) = .017, *p* = .983, η^2^ = .001. There were also no significant interactions between the experimental conditions and BMI, *F*(2, 56) = .668, *p* = .517, η^2^ = .022 (BMI was not reported by two individuals), gender *F*(2, 60) = 1.496, *p* = .232, η^2^ = .046, the length of relationship, *F*(2, 60) = .559, *p* = .575, η^2^ = .017, or whether both members of the couple were tested, *F*(2, 60) = .139, *p* = .871, η^2^ = .004.

To examine whether there was a relationship between anxious or avoidant attachment styles and the changes in IAcc in different conditions, a correlational analysis was carried out. Given that anxious and avoidant attachment scores, as measured with the Experience in Close Relationships–Revised Questionnaire, are often closely correlated, we used partial correlations to control for the contribution of one attachment style while investigating the contribution of the other. Neither attachment measure correlated with IAcc_change_ scores; there was no significant link between anxiety scores and IAcc_change_ in the self condition, *r*(29) = −.083; *p* = .659, or the partner condition, *r*(29) = −.079; *p* = .674, while controlling for avoidance, and neither was there a significant link between avoidance scores and IAcc_change_ in the self condition, *r*(29) = −.257, *p* = .162, or partner condition, *r*(29) = −.221, *p* = .233, when controlling for anxiety.

## Discussion

The awareness of internal bodily sensations, termed *interoception*, plays a crucial role in self-awareness. Researchers have shown previously that IAcc can be increased when one’s attention is directed to self-related information, such as a photograph of one’s face. However, these investigations only examined the self in isolation, or in the context of unknown people. Importantly, the experience of the self changes markedly depending on the social context, and the real or imagined presence of significant others, such as romantic partners, evokes a “relational self” distinct from that active in other more individualistic contexts. In particular, romantic partners have a central role in shaping one’s self-perception as they act as a social mirror for one’s self (Andersen & Chen, 2002; Aron & Nardone, 2011; Drigotas, Rusbult, Wieselquist, & Whitton, 1999).

The present study investigated whether partner-face observation would enhance one’s IAcc in a relational context, similarly to the effect of self-face observation in an individual context. During a heartbeat counting task, concurrent partner-face observation improved accuracy in the low baseline IAcc group, but self-face observation did not improve accuracy in the heartbeat counting task. Our results suggest that focusing on a romantic partner does indeed increase interoceptive processing. Furthermore, in a relational context, the partner seems to function as a more relevant cue to interoceptive awareness than self-face observation. Surprisingly, the effect of viewing one’s partner has on IAcc was not moderated by individual differences in attachment, suggesting that this effect functioned on a more fundamental, embodied level that was not sensitive to higher level cognitive and motivational representations.

The finding that individuals with initially poor IAcc showed a significant improvement during partner-face observation, but not during self-face observation might initially seem surprising as past studies have demonstrated that self-observation increases IAcc (Ainley et al., 2012, 2013; Maister & Tsakiris, 2014); however these studies presented the self compared with unfamiliar others or in isolation, whereas in our study we placed the self in a relational context by featuring pictures of romantic partners during the task.

Taylor and colleagues’ (2009) imaging findings support the existence of a special self-processing in the presence of the partner, which can explain the context-dependent differences in the fluctuation of IAcc during self-face observation. Brain areas associated with interoception, such as the insula (Craig, 2010; Pollatos et al., 2007), have also been associated with both the recognition of one’s own face (Morita et al., 2008) and the partner’s face (Bartels & Zeki, 2000; Fisher et al., 2005; Ortigue, Bianchi-Demicheli, Hamilton, & Grafton, 2007). However, it is interesting to note that the studies reporting insula activity in response to the self-face generally included the presentation of unfamiliar faces in the task as a comparison condition (e.g., Morita et al., 2008). In contrast, when the self-face was presented in a sequence including the face of a romantic partner instead of an unfamiliar other, the insula showed activation only during the recognition of the partner’s face and not of the self-face (Taylor et al., 2009). This later finding may lend indirect support to our suggestion that partner-related stimuli become more relevant to interoceptive awareness than self-related stimuli when the self is perceived in a relational context. An interesting future study would be to investigate interoceptive awareness twice in the same participants, once in which the self-face is presented interspersed with an unfamiliar face (to elicit an “individual” self-representation) and once in which presentation of the self-face is interspersed with the romantic partner’s face (to elicit a “relational” self-representation). Thus, the effects of the two self-contexts on IAcc could be directly compared within-subjects, both on both a behavioral and neurological basis.

Despite finding significant effects of partner-face observation on IAcc, we found no moderating effects of attachment style. Although some studies suggest that insecure attachment can hamper one’s emotional and stress regulation (Diamond et al., 2008; Feeney & Kirkpatrick, 1996; Levenson & Gottman, 1983; Saxbe & Repetti, 2010), and the ability to regulate the experience of pain in the presence of one’s partner (Krahé et al., 2015), definitive studies have not yet been conducted that directly focused on the relationship between adult attachment styles and interoceptive awareness. In our study, we did not find any relationship between attachment styles and interoceptive awareness, either as a trait ability at baseline, or in response to partner-focus. This finding is consistent with a number of other studies which have failed to find any links between explicitly measured adult attachment styles and more implicit and affective processing of the partner (e.g., Banse, 1999). However, we can draw no firm conclusions from the nonsignificant correlation involving attachment in this study because of our relatively small sample size and potential lack of adequate power. Further research is needed to precisely elucidate the relationship between attachment styles and embodied self-awareness in relational and nonrelational contexts.

Researchers have shown that the self-regulation of one’s emotions and stress change substantially depending on whether a romantic partner or an image of the partner is present (Diamond et al., 2008; Master et al., 2009; Younger, Aron, Parke, Chatterjee, & Mackey, 2010). Furthermore, there is often also a strong coregulation between romantic partners, whereby one’s own and one’s partner’s affective and physiological responses are linked, which can contribute to both partners’ emotional stability (Butler & Randall, 2013). Self-regulatory behaviors are thought to form an important part of the relational self, which is activated not only when a romantic partner is present, but also in situations where a reminder of the partner—such as a picture—is presented (Andersen & Chen, 2002). Given this activation, and the known role of interoception in emotional regulation (e.g., Füstös et al., 2013), our finding of increased IAcc during partner-face observation may provide the underlying mechanism by which self-regulation of arousal and emotions is increased in the presence of the partner.

An interesting finding of the current study was that partner-face observation had a greater enhancing effect on IAcc than did self-face observation. Because we presented self-face and partner-face trials in an intermixed order rather than using a blocked design, a relational context was evoked across the entire task. Although self-images and partner-images are both relevant sources of exteroceptive information for the self, the romantic partner’s face may be more relevant than one’s own face to the currently experienced self in a relational context. In everyday life, the partner’s reactions to the self are of great importance, as they function as a social mirror for one’s self, reflecting those signals that are currently most salient for the relationship (e.g., Aron & Nardone, 2011; Andersen & Chen, 2002; Drigotas et al., 1999). Thus, this enhancement of interoceptive processing when focusing on the partner may be an important mechanism to protect the stability of the relationship.

In conclusion, the present study extended previous findings demonstrating the enhancing effects of self-observation on IAcc (Ainley et al., 2012, 2013; Maister & Tsakiris, 2014) in response to partner-face observation in a relational context. We found that participants with low baseline IAcc showed the greatest improvement in the heartbeat counting task (Schandry, 1981) during partner-face observation, which suggests a distinct, embodied self-processing in the presence of the partner. It is likely that romantic partners act as social mirrors by providing exteroceptive information regarding the self, which may be even more relevant in a relational context than the signals arising from self-observation. We suggest that this exteroceptive information in a relational context may be the mechanism underlying the increased self-regulation of emotional and stress responses when in the presence of romantic partners, and thus may have important implications for our understanding of embodied self-awareness in romantic relationships.

## Figures and Tables

**Table 1 tbl1:** Descriptive Statistics of Interoceptive Accuracy (IAcc) Scores and Heart Rate for Lower (Below Median IAcc) and Higher (Above Median IAcc) Groups

	Values of IAcc			Values of heart rate		
Group	*M*	*SD*	95% CI	*M*	*SD*	95% CI
Lower IAcc (*n* = 16)						
Blank screen	.47	.09	[.42, .51]	89.28	16.04	[80.73, 97.83]
Self-face	.48	.11	[.42, .54]	90.06	16.31	[81.37, 98.75]
Partner-face	.53	.09	[.48, .58]	89.53	15.97	[81.02, 98.04]
Higher IAcc (*n* = 16)						
Blank screen	.74	.13	[.67, .81]	80.13	13.08	[73.16, 87.1]
Self-face	.70	.15	[.62, .78]	79.49	11.62	[73.29, 85.68]
Partner-face	.72	.12	[.66, .79]	80.01	12.61	[73.32, 86.77]
*Note*. CI = confidence interval.
